# Immature B Cell Egress from Bone Marrow Is SOCS3 Independent

**DOI:** 10.1371/journal.pone.0136061

**Published:** 2015-08-14

**Authors:** Kristina Nadrah, Thomas C. Beck, João P. Pereira

**Affiliations:** Department of Immunobiology, Yale University School of Medicine, New Haven, Connecticut 06520, United States of America; The University of Melbourne, AUSTRALIA

## Abstract

Suppressor of cytokine signaling (SOCS)-3 has been suggested to regulate CXCR4 signaling in a variety of human cell lines. In mice, conditional SOCS3 inactivation in hematopoietic cells including B-lineage lymphocytes has been reported to exacerbate CXCR4-signaling and focal adhesion kinase phosphorylation, which resulted in altered immature B cell distribution in bone marrow (BM) due to sustained α4β1 integrin-mediated adhesion to the extracellular matrix. However, a recent study examining conditional SOCS3 deletion specifically in B-lineage cells failed to detect significant roles in B-lineage cell retention in BM. In this study we carefully examined the role played by SOCS3 in CXCR4 signaling in developing B cell subsets. We show that in mice conditionally deficient in SOCS3 exclusively in B cells (*Socs3* ^*fl/fl*^ *Mb1* ^*cre/+*^) there was no detectable difference in B cell development in BM and in periphery. We show that SOCS3 deficient and sufficient immature B cell subsets are similarly distributed between BM parenchyma and sinusoids, and are equally competent at exiting BM into peripheral blood. Furthermore, we found no significant differences in CXCR4 desensitization upon ligand exposure in developing B lymphocyte subsets. Consequently, SOCS3-deficient and sufficient B-lineage cell migration towards CXCL12 *in vitro* was undistinguishable, and B-lineage cell amoeboid motility within BM parenchyma was also unaffected by SOCS3-deficiency. Thus we conclude that SOCS3 has no detectable influence on biological processes known to be controlled by CXCR4 signaling.

## Introduction

B lymphocytes develop in bone marrow (BM) through sequential stages characterized by the differential expression of several cell surface receptors. At the proB and preB cell stages, B-lineage cells undergo somatic recombination of immunoglobulin heavy and light chain V(D)J genes. Productive gene rearrangements result in the expression of a functional B cell receptor (BCR) on the cell surface and developmental transition to the immature B lymphocyte stage. Although small numbers of essentially all B cell subsets can be found in blood and in the periphery of normal mice, it is at the immature B lymphocyte stage that cells become competent for exiting BM [1]. In general, lymphocytes are strictly dependent on Sphingosine 1-phosphate (S1P) and S1P receptor-1 for exiting thymus (for T cells) and lymph nodes (T and B cells), such that defects in S1PR1 or in S1P production result in a ~ 50–1,000 fold reduction in peripheral lymphocytes [2]. However, immature B lymphocytes rely little on the egress-promoting activity of S1PR1 and S1P given that pharmacological or genetic deficiency in either molecule reduces immature B cell export from BM by 2–3 fold only [1, 3]. Remarkably, immature B lymphocytes, and other hematopoietic cells, depend minimally on Gαi protein-coupled chemoattractant receptors for exiting BM, when compared to T cells and their dependency on Gαi protein signaling for thymic egress [4, 5]. Instead, hematopoietic cells, and particularly immature B lymphocytes, are highly sensitive to passive (cell extrinsic) mechanisms enforcing cell exit from BM, such that egress is mostly controlled by attenuation of BM retention operated by CXCR4 signaling [5]. In developing B cell subsets, CXCR4 is expressed at highest amounts at the proB cell stage, and its expression reduces progressively in subsequent developmental stages [6–8]. At the immature B lymphocyte stage, cells can be further retained inside BM sinusoids through the activity of two chemoattractant receptors, namely Cannabinoid receptor 2 and Sphingosine 1-phosphate (S1P)-receptor 3 before exiting BM [8, 9]. Importantly, CXCR4 expression is further reduced by 2-fold in immature B cell subsets located in sinusoids, and antagonizing CXCR4 downregulation is sufficient for blocking egress BM [5]. BCR signaling prevents CXCR4 downregulation in immature B cell subsets, and promotes their retention in BM parenchyma [5]. However, whether additional mechanisms control CXCR4 downregulation remains incompletely understood.

Upon binding to its ligand CXCL12, CXCR4 signals predominantly through interactions with Gi and Gq proteins that result in activation of G protein coupled receptor related kinases followed by receptor internalization and desensitization [10–14]. CXCR4 internalization (or desensitization) is critical for appropriate regulation of CXCR4 signaling, given that defects in its internalization maintain the receptor in a constitutively active form that causes an immune deficiency syndrome named Warts, Hypogammaglobulinemia, Infections and Myelokathexis (WHIM) syndrome in humans [15–18]. WHIM patients exhibit reduced lymphocyte and granulocyte numbers in peripheral blood, while these cells are overrepresented in BM. Importantly, antagonizing CXCR4 signaling in WHIM patients results in the mobilization of granulocytes and B lymphocytes from BM into peripheral blood circulation [19].

Early studies identified SOCS3 (suppressor of cytokine signaling 3) protein as an important regulator of CXCR4 signaling in the IM-9 B cell line (Soriano et al., 2002). Furthermore, SOCS3 was demonstrated to associate with CXCR4 protein by immunoprecipitation, suggesting that SOCS3 may directly affect CXCR4 signaling (Soriano et al., 2002). Overexpression of SOCS3 in IM-9 B cells impaired CXCR4 mediated chemotaxis towards SDF-1 in vitro (Soriano et al., 2002). Whether SOCS3 is influencing CXCR4 signaling directly or indirectly remained unclear. In vivo, SOCS3 expression increases from the pro-B cell to immature B cell stages of development, and conditional SOCS3 deletion in developing B cell subsets (using a mouse mammary tumor virus (MMTV)-cre transgene, which is also active in epithelial cells, megakaryocytes and erythroid cells) led to an egress defect of immature B cells from BM (Le et al., 2007). It was proposed that SOCS3 negatively regulates CXCL12-induced focal adhesion kinase phosphorylation and ubiquitination, and, thus, SOCS3 signaling reduces CXCR4-dependent integrin-mediated adhesion [20]. Furthermore, growth hormone induction of SOCS3 expression in hematopoietic precursors leads to their mobilization into peripheral blood (Pello et al., 2006). These data together suggest SOCS3 plays a role in mediating the release of immature BM B cells into the periphery. However, another study examining SOCS3 function in B cell subsets failed to detect B cell developmental and recirculatory defects in mice conditionally deficient in SOCS3 expression in B cells (using Mb1-cre mice), nor when SOCS3 was deleted in MMTV-cre active cells [21]. Thus, whether SOCS3 functions to control developing B cell positioning and retention in BM remains controversial.

Here we examined the role played by SOCS3 in developing B cell movement and retention within BM parenchyma by conditional SOCS3 inactivation in developing B cells using the Mb1-cre allele. Our studies show unambiguously that SOCS3 is not required for developing B cell motility and retention in BM parenchyma as well as access to BM sinusoids. Furthermore, we found no evidence sustaining a role played by SOCS3 in the control of CXCR4-mediated migration, nor in its desensitization upon exposure to CXCL12.

## Materials and Methods

### Mice

Adult C57BL/6 (CD45.2^+^) mice, adult Boy/J (CD45.1^+^; 002014) mice were obtained from The Jackson Laboratory. *Socs3* ^*fl/fl*^ mice were from The Jackson Laboratory; *Mb1* ^*cre/+*^ and *Rag1* ^*GFP/+*^ mice were from our internal colony. *Cxcr4* ^*fl/fl*^ and *Socs3* ^*fl/fl*^ mice were crossed with *Mb1* ^*cre/+*^ mice to generate *Socs3* ^*fl/fl*^ *Mb1* ^*cre/+*^ and *Cxcr4* ^*fl/fl*^ *Mb1* ^*cre/+*^ mice. *Socs3* ^*fl/fl*^ *Mb1* ^*cre/+*^ mice were bred to *Rag1* ^*GFP/+*^ mice to generate *Socs3* ^*fl/fl*^ *Mb1* ^*cre/+*^ Rag1^GFP/+^ mice. All mice were cared for in accordance with institutional animal care and use committee approved protocols from Yale University School of Medicine.

### Tissue collection, cell stains, and flow cytometry

Mice were sacrificed by CO2 inhalation, and whole blood was immediately collected in EDTA-coated tubes. Red blood cells in whole blood were lysed with lysis buffer prior staining. Tibias and femurs were surgically removed and bone marrow cells flushed with collection media (DMEM supplemented with 2% FBS, 50 IU/ml penicilin, 50ug/ml streptomycin.) Spleens were minced in collection media and filtered through cell strainers. Single cell suspensions were stained with fluorescently labeled antibodies and DAPI. The following antibodies were used: PerCpCy5.5-anti-B220 (RA3-6B2),PE-Cy7-anti-IgM (II/41),biotin-anti-IgD (11–26), APC-anti-CD93 (AA4.1) (all eBioscience), PE-anti-IgD (11-26c.2a), Alexa Fluor 700-anti-CD45.2 (104), Pacific Blue- anti-CD45.1 (A20) (all Biolegend), biotin-anti-CXCR4, PE-anti-CD19 (1D3) (all BD Biosciences) and, Qdot 605-streptavidin (Invitrogen). Flow cytometry analyses were performed on a LSR II (BD Biosciences) or FACSCalibur and data analyzed by FlowJo version 9.2 (Tree Star, Inc).

### Labeling of sinusoidal B cell subsets

B-lineage cells retained inside BM sinusoids were analyzed as previously described [8]. Briefly, sinusoidal cells were labeled by tail vein injection of mice 1.5 μg phycoerythrin-conjugated rat anti–mouse CD19 in 200μL of sterile PBS and then killed in CO_2_ chamber after 2 min.

### Mixed bone marrow chimeras

Bone marrow cells were isolated from donor mice (congenic CD45.1 and CD45.2 mice) by flushing femurs and tibias with collection media. A total of 3x10^6^ BM cells were transferred intravenously into lethally irradiated mice. BM transplant recipient mice were analyzed 6–8 weeks after reconstitution.

### CXCR4 re-sensitization and de-sensitization, and cell migration assays

BM cells were resuspended in chemotaxis buffer consisting of 0.5% fatty acid free BSA, 10mM HEPES, 2mM L-glutamine, and 1% Pen/Strep, to a concentration of 10^7^cells/ml. For re-sensitization, cells were incubated for 1h at 37°C. Approximately 10^6^ cells were subsequently exposed to various amounts of CXCL12 for 30 min at 37°C, then washed with ice-cold buffer (2% BSA, 1mM Na_2_EDTA, 0.1%NaN_3_ in 1xPBS) and stained. Chemotaxis assays were performed using 1x10^6^ BM or spleen cells incubated for 30 minutes with 1x DMEM (Cellgro) containing 0.5% fatty acid free BSA (EMD Biosciences), 5% of antibiotics (Cellgro), L-glutamine (Cellgro), and HEPES (Cellgro). Cells were allowed to migrate through 5μm pore-sized transwells (Corning) towards soluble CXCL12 (R&D) for 3 hours at 37 C. Cells were collected, stained, and analyzed by flow cytometry.

### PCR and QPCR

Total RNA was extracted with an Rneasy Mini Kit (Qiagen) and cDNA sythesized from up to 5μg of RNA using SuperScript III Reverse Transcriptase (Life technologies). Nucleic acids were quantified by spectrophotometry using NanoDrop 2000 (Thermo Scientific). Primers used for QPCR: SOCS3 reverse: 5'–AGC TGG GTC ACT TTC TCA TA– 3' SOCS3 forward: 5'–TAC TGA GCC GAC CTC TCT C– 3'. Real-time quantitative PCR (QPCR) was performed using SensiFAST SYBR Lo-ROX Kit (Bioline). Relative *Socs3* expression was normalized for hypoxanthine-guanine phosphoribosyltransferase (*Hprt*) expression.

### Intravital 2-photon microscopy

Mice were anesthetized with a cocktail of ketamine/xylazine and calvaria bone plates surgically exposed, as described [22]. Mouse blood vessels were visualized after intravenous injection of tetramethylrhodamine conjugated dextran (MW 2,000 KDa). Deep-tissue image acquisition were performed using a LaVision TriM Scope II (LaVision Biotec) microscope equipped with a Chameleon Vision II (Coherent) laser. The laser beam (875nm) was focused through an Olympus water immersion lens (20X). The X/Y scanned area was 400 x 400 μm, and the Z axis was between 39–50μm acquired with 3 μm Z-steps. Serial optical sections were acquired every 20 second intervals, for 30 minutes. Statistical analyses of cell movement and amoeboid cell shapes were performed as previously described [5].

## Results

### Immature B cell egress from bone marrow is SOCS3 independent

**Fig 1:**
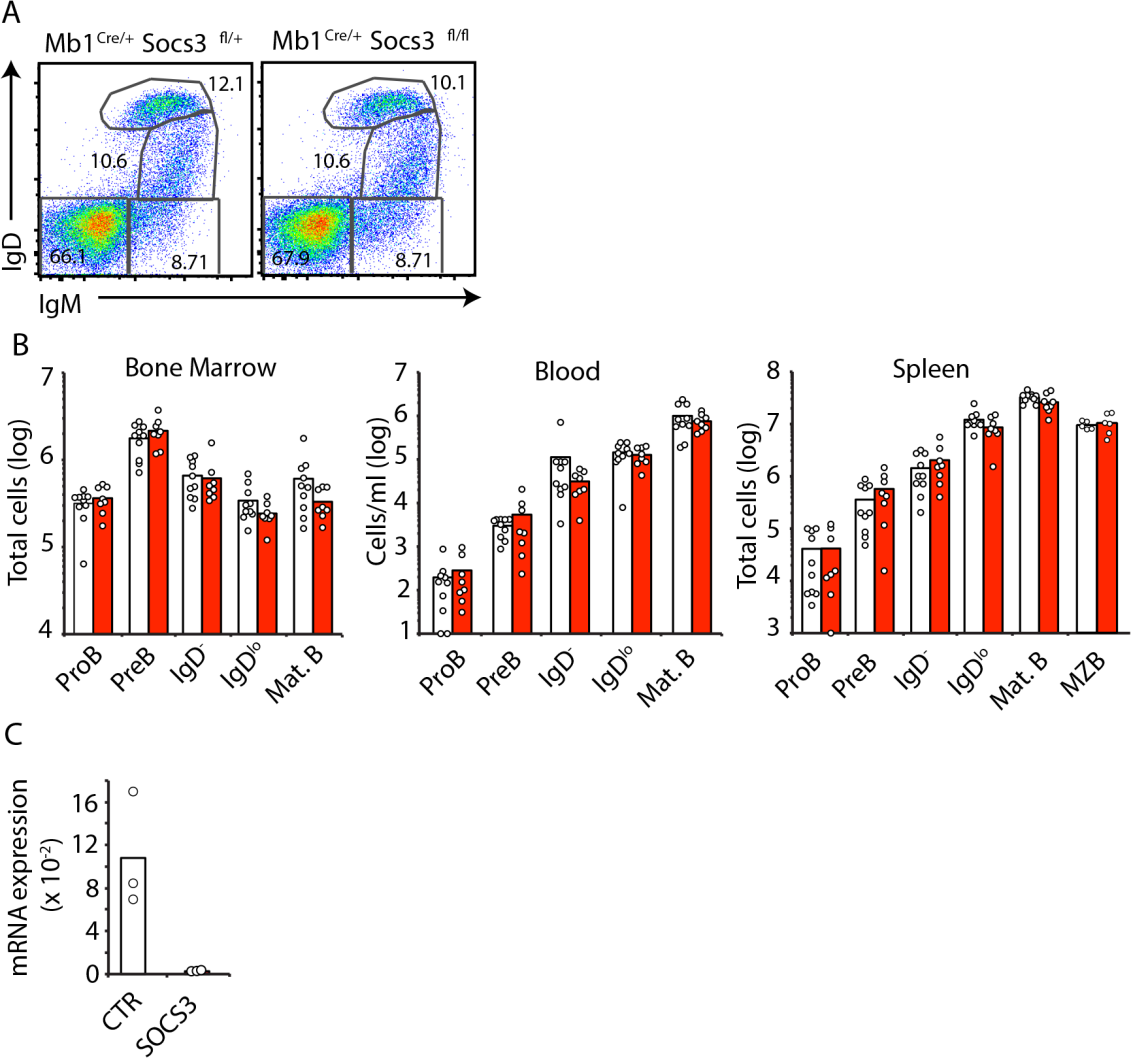
B cell development is independent of SOCS3 signaling. **A**, Developing B cell subsets in BM examined by IgM and IgD cell surface expression by flow cytometry. Cells were previously gated as live (DAPI^-^) CD19^+^ cells. **B**, Enumeration of developing B cell subsets in BM (left), blood (center) and spleen (right) of *Socs3* ^*+/+*^ (white) or *Socs3* ^*fl/fl*^ (red) *Mb1* ^*Cre/+*^ mice at 8–10 weeks of age. Bars indicate average, circles depict individual mice. Data were pooled from 4 independent experiments. C, *Socs3* expression in proB cells from *Socs3* ^*+/+*^ (white) or *Socs3* ^*fl/fl*^ (red) *Mb1* ^*Cre/+*^ mice. Expression is relative to *Hprt*. Bars indicate average, circles depict individual mice. ns (not significant; unpaired two-tailed student’s *t* test).

**Fig 2:**
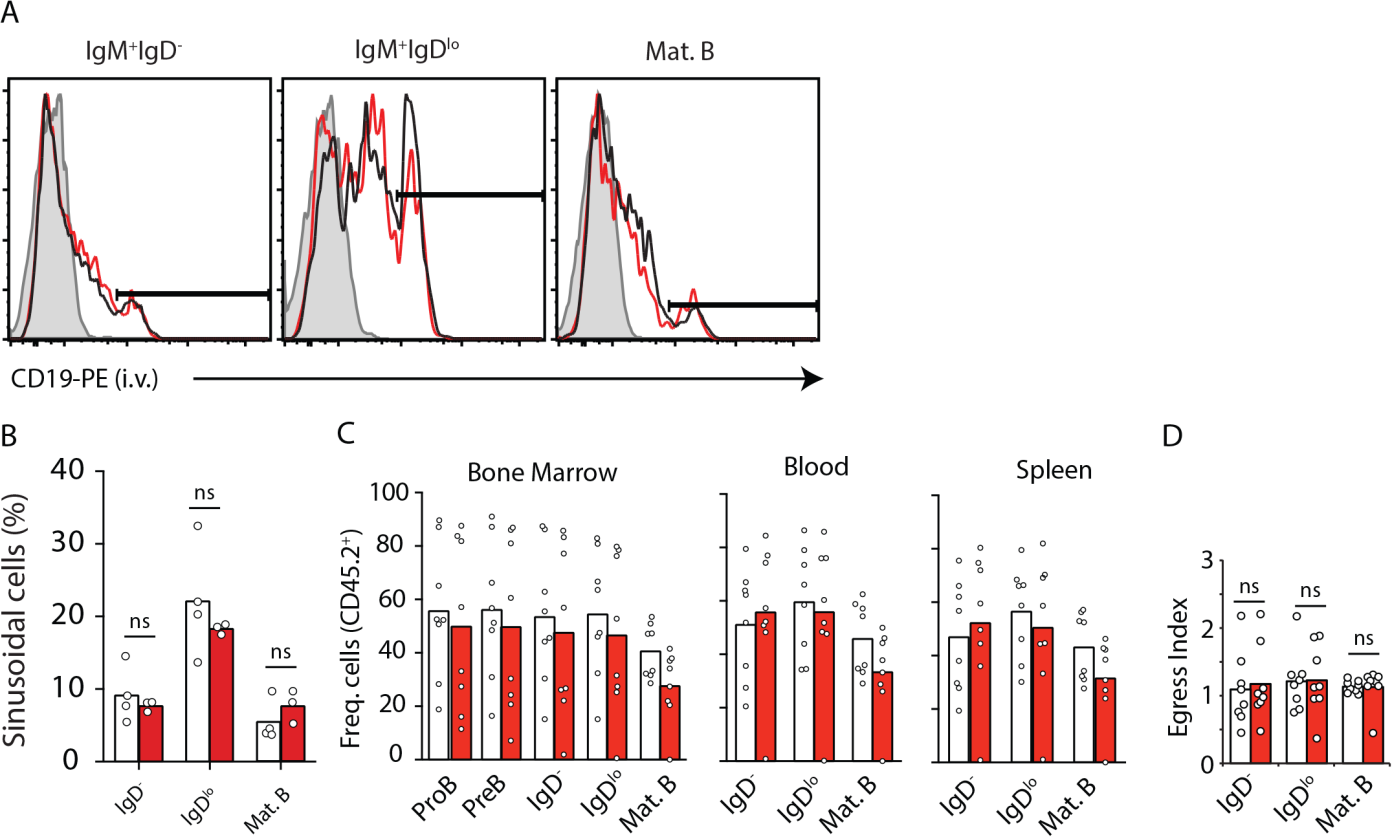
B-lineage cell positioning in parenchyma and sinusoids, and egress from BM is independent of SOCS3 signaling. **A**, Distribution of immature IgM^+^ IgD^-^ and IgM^+^ IgD^lo^ (both CD93^+^) cells in BM parenchyma (CD19-PE^-^) and in sinusoids (CD19-PE^+^). Histogram of CD19-PE injected i.v. into *Socs3*-deficient (red) and sufficient (black) mice 2 minutes prior to sacrifice. Filled histogram shows background fluorescence in the indicated B cell subset from a mouse that did not receive CD19-PE. **B**, Enumeration of immature B cell subsets in BM parenchyma and sinusoids of *Socs3*-deficient (red) and sufficient (white) mice. Bars indicate average, circles depict individual mice. Data were pooled from 3 independent experiments. **C**, Frequency of CD45.2^+^ B-lineage cell subsets in BM (left), blood (middle), and in spleen (right) of lethally irradiated WT mice reconstituted with 50% CD45.2^+^ *Socs3* ^*f/f*^ *Mb1* ^*Cre/+*^ (red) or *Socs3* ^*+/+*^ *Mb1* ^*Cre/+*^ (white) BM cells mixed with 50% CD45.1^+^ WT BM cells. **D**, Egress index of B cell subsets from BM calculated as the ratio of CD45.2^+^ cells in blood by BM. Bars indicate average, circles depict individual mice. Data is representative of three independent experiments. ns (not significant; unpaired two-tailed student’s *t* test).

In order to determine if SOCS3 plays a role in mediating the release of immature BM B cells into the periphery we examined the distribution of developing B cell subsets in BM, blood and spleen of mice conditionally deficient in SOCS3 expression in B lineage cells. To this purpose we crossed *Socs3* ^*fl/fl*^ mice with *Mb1* ^*Cre/+*^ mice in which cre recombinase expression is highly efficient from the proB cell stage [23]. In contrast to findings of Silberstein and colleagues [20], and in agreement with findings reported by Tarlinton and colleagues [21] we observed that SOCS3 deficient and sufficient B cell subsets were present in BM at comparable frequencies (**Fig 1A**). Furthermore, we did not observe a statistically significant numerical difference between SOCS3 deficient and sufficient B cell subsets across BM, blood, and spleen (**Fig 1B**), despite the fact that *Socs3* expression was undetectable in B cells from *Socs3* ^*fl/fl*^ *Mb1* ^*Cre/+*^ mice, while it was readily detectable in B cells from littermate controls (**Fig 1C**). Although these data suggested SOCS3 is not required for developing B cell subset circulation, we considered the possibility that SOCS3 might play a subtle role in B cell retention in BM. As developing B cell subsets are temporarily retained inside BM sinusoids before exiting into peripheral blood [8, 24], the size of sinusoidal B cell population can be used as a sensitive indicator of altered BM egress. Thus, we examined the positional distribution of developing B cells deficient and sufficient in *Socs3* in BM parenchyma and sinusoids. To distinguish between parenchymal and sinusoidal immature B cells we injected mice intravenously with CD19 antibody conjugated to phycoerythrin for 2 minutes prior to sacrifice [8]. We found that SOCS3-deficient B-lineage cells were similarly represented in BM parenchyma and sinusoids (**Fig 2A and 2B**). To further exclude a minor role in B cell retention in BM, we examined SOCS3 deficient B cells in direct competition with WT B cells using a 50:50 mixed BM chimera approach. Even in competition with SOCS3 sufficient B cells, we observed that SOCS3 deficient B cells were present across BM, blood, and spleen in similar frequencies (**Fig 2C**). To rule out the possibility that SOCS3 plays a subtle role in B cell retention in BM we analyzed the BM egress index of immature and mature B cells. To this purpose we divided the frequency of CD45.2+ immature B and mature B cell subsets in peripheral blood by the frequency of CD45.2+ immature B and mature B cell subsets in bone marrow (**Fig 2D**). We found no evidence of altered B-lineage cell retention in BM.

### CXCR4 expression and desensitization is independent of SOCS3 signaling in developing B cells

**Fig 3:**
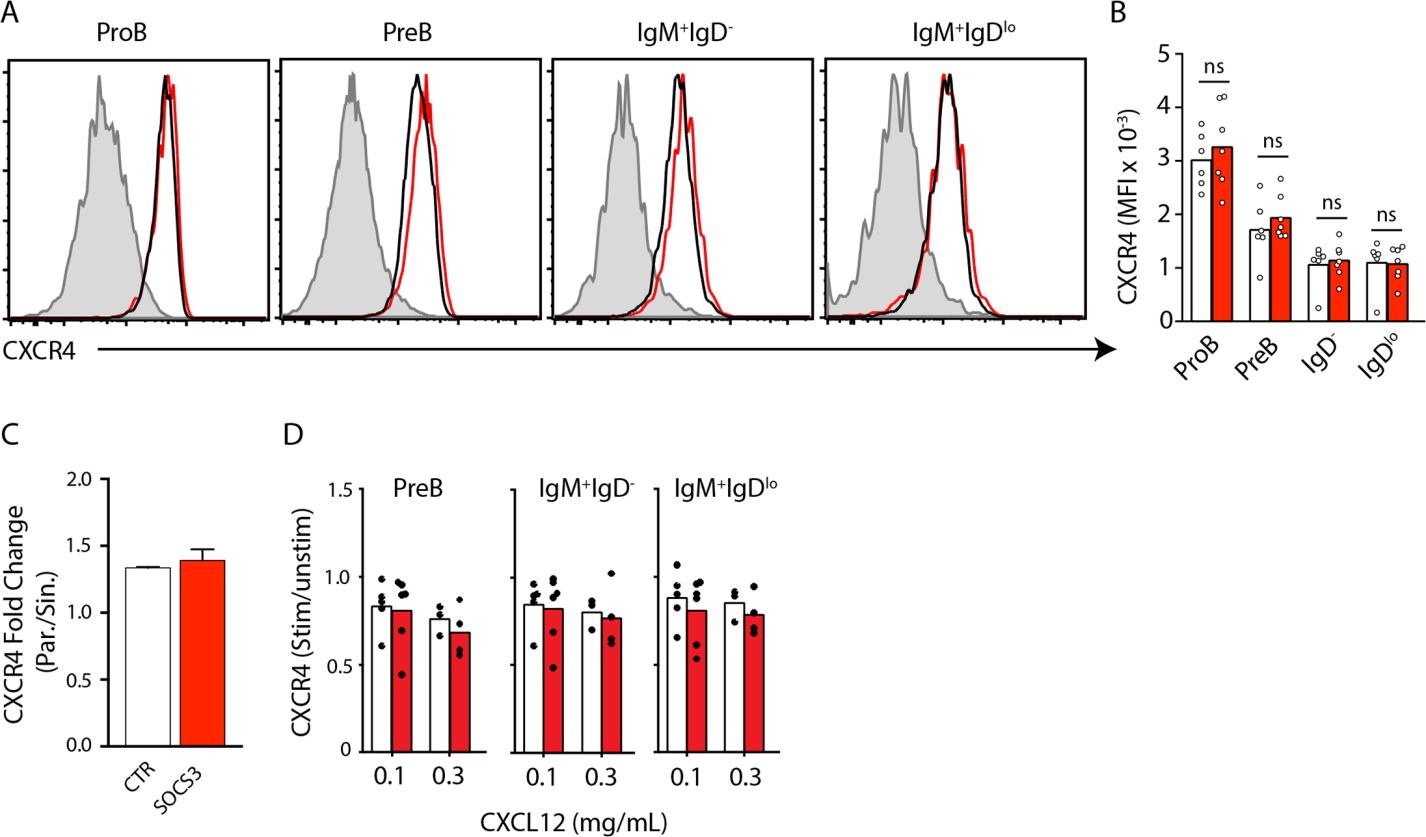
CXCR4 expression and desensitization are independent of SOCS3 signaling in B-lineage cells. **A**, CXCR4 expression in developing B cell subsets in BM of *Socs3* ^*fl/fl*^ (red) or *Socs3* ^*+/+*^ (black) *Mb1* ^*Cre/+*^ mice. **B**, Mean fluorescence intensity (MFI) of CXCR4 expression on the surface of developing B cell subsets isolated from BM of *Socs3* ^*fl/fl*^ (red) or *Socs3* ^*+/+*^ (white) *Mb1* ^*Cre/+*^ mice. Bars indicate the mean, circles depict individual mice analyzed. Data was pooled from 3 independent experiments. **C**, Fold change in CXCR4 expression in IgM^+^IgD^lo^ cells distributed in BM parenchyma and sinusoids. White, *Socs3* ^*+/+*^ *Mb1* ^*Cre/+*^; Red, *Socs3* ^*fl/fl*^ *Mb1* ^*Cre/+*^ mice. Bars indicate average and standard deviation. **D**, Change in MFI of CXCR4 surface expression on B lineage cell subsets from *Socs3* ^*+/+*^ *Mb1* ^*Cre/+*^ (white) or from *Socs3* ^*fl/fl*^ *Mb1* ^*Cre/+*^ (red) mice stimulated with CXCL12 at the indicated concentrations. Data shown is the ratio between CXCR4 MFI in unstimulated cells and after stimulation. Bars indicate the mean, circles depict individual mice analyzed. Data was pooled from 3 independent experiments.

CXCR4 is a critical chemoattractant receptor that signals the homing and retention of multiple hematopoietic stem, progenitor, and differentiated cell subsets [25]. Upon engaging its ligand, the cytoplasmic tail of CXCR4 is phosphorylated and desensitized by beta-arrestin-mediated receptor internalization. Defects in CXCR4 desensitization result in sustained CXCR4 signaling, which increases cell retention in BM and causes peripheral cytopenia [15, 17, 26]. In IM-9 and HEK293T cell lines, CXCL12 binding to CXCR4 increased SOCS3 expression through the JAK/STAT pathway. SOCS3 then subsequently complexes with CXCR4 and attenuates its ability to signal by regulating JAK/STAT activation [27]. As SOCS3 can complex with CXCR4, presumably with its cytoplasmic tail, and was proposed to control B cell retention in BM, we hypothesized that SOCS3 might control CXCR4 surface expression by interfering with its desensitization in developing B cells in BM. To test this hypothesis, we first assessed the surface expression of CXCR4 on SOCS3 deficient and sufficient developing B cell subsets in BM. We observed no statistically significant difference in the expression of CXCR4 between SOCS3 sufficient and deficient B-lineage cells (**Fig 3A and 3B**). Our previous work showed that as immature B cells transit from parenchyma to sinusoids, CXCR4 surface expression is reduced by approximately two-fold [5]. Moreover, immature B cell egress was inhibited by overexpression of a desensitization mutant form of CXCR4 [5]. Therefore, we next measured CXCR4 expression on SOCS3 deficient and sufficient immature IgD^lo^ B cell subsets in BM parenchyma and sinusoids. We found that SOCS3 deficient and sufficient immature B cells downregulated CXCR4 similarly (**Fig 3C**). Furthermore, we resensitized BM cells from *Socs3* ^*fl/fl*^ or *Socs3* ^*+/+*^ *Mb1* ^*Cre/+*^ mice and pulsed them with media alone or with CXCL12 at increasing concentrations (0.1**μ**g/ml and 0.3**μ**g/ml). We found significant CXCL12-mediated CXCR4 internalization at all concentrations and in all B cell subsets (not shown), and desensitization occurred with similar efficiency in SOCS3-deficient and sufficient B-lineage cell subsets (**Fig 3D**). Combined, these data exclude a significant role played by SOCS3 signaling in CXCR4 expression and desensitization in B-lineage cells.

### CXCR4-mediated B cell motility is independent of SOCS3 signaling

**Fig 4:**
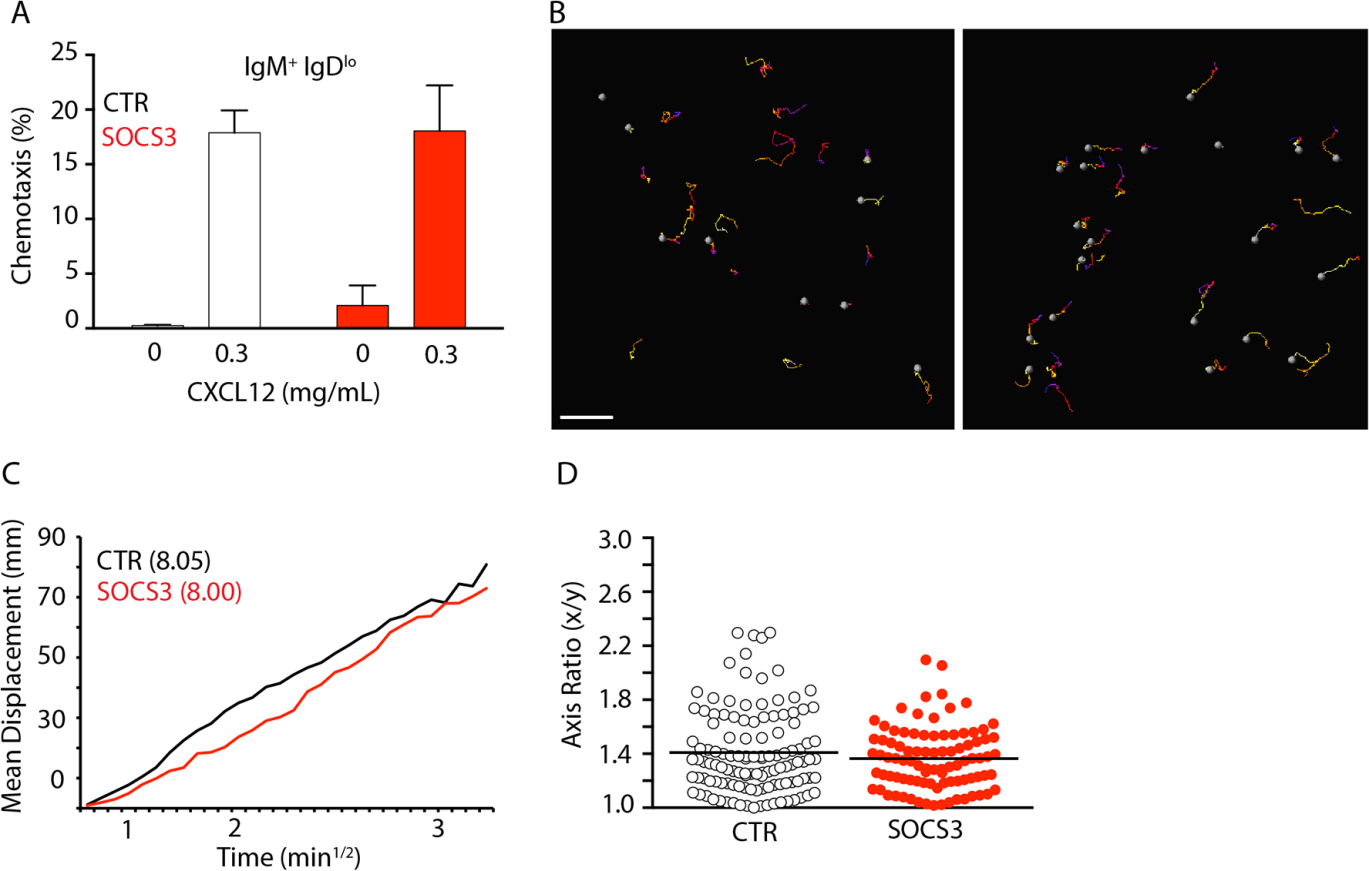
CXCR4-mediated B-lineage cell migration is independent of SOCS3 signaling. **A**, Transwell migration assay of immature B cells from *Socs3* ^*fl/fl*^ (SOCS3, red) or *Socs3* ^*+/+*^ (CTR, white) *Mb1* ^*Cre/+*^ mice towards CXCL12 in vitro. Bars indicate average and standard deviation. **B**, Distribution of GFP^+^ B lineage cells in BM of *Socs3* ^*fl/fl*^ *Mb1* ^*Cre/+*^ mice (left) and *Socs3* ^*+/+*^ *Mb1* ^*Cre/+*^ (right) mice. Movement of GFP^+^ cells tracked for 30 minutes. Colored lines depict cell trajectories over time. Scale bar is 50 μm. **C**, Mean motility coefficient of B-lineage cells from *Socs3* ^*fl/fl*^ *Mb1* ^*Cre/+*^ mice (red) and *Socs3* ^*+/+*^ *Mb1* ^*Cre/+*^ (black) mice. Cell displacement from starting coordinates is plotted against the square root of time. **D**, Measurement of cell axis ratio of GFP+ B lineage cells from *Socs3* ^*fl/fl*^ *Mb1* ^*Cre/+*^ mice (red) and *Socs3* ^*+/+*^ *Mb1* ^*Cre/+*^ (black) mice. Lines indicate mean, circles depict individual cells analyzed.

Previous studies demonstrated that SOCS3 regulates CXCR4-mediated chemotaxis towards CXCL12 [27]. Although SOCS3 deficiency did not affect CXCR4 expression and/or internalization it is possible that SOCS3 signaling interferes with CXCR4-mediated chemotaxis. To address this hypothesis we isolated and resensitized BM cells from *Socs3* ^*fl/fl*^ or *Socs3* ^*+/+*^ *Mb1* ^*Cre/+*^ mice and performed transwell migration assays towards CXCL12. We observed no statistical difference between SOCS3 deficient or sufficient immature B cells migration towards CXCL12 in vitro (**Fig 4A**). Using intravital 2-photon microscopy, we previously reported that CXCR4 controls B-lineage cell movement in BM, and B-lineage cell motility correlates with BM retention [5]. Although SOCS3 deficiency did not alter B-lineage cell retention in BM it is possible that SOCS3 signaling affects B cell migration in vivo. Cell migration can be quantified in multiple ways. The mean displacement plot is particularly informative because it may reveal whether cells move randomly within tissues, or whether their movement is directional (e.g from parenchyma to sinusoids) or confined [28]. Importantly, when cells move randomly, displacement plots allow measuring of how quickly cells displace from their starting positions (motility coefficient). To determine if SOCS3 influenced B-lineage cell migration in vivo we examined SOCS3-deficient and sufficient B-lineage cells by two-photon microscopy of the calvaria BM of *Socs3* ^*fl/fl*^ or *Socs3* ^*+/+*^ *Mb1* ^*Cre/+*^ *Rag1* ^*GFP/+*^ mice. We found that SOCS3-deficient and sufficient B-lineage cells moved randomly and with similar motility coefficients (8.00 and 8.05, respectively) in BM parenchyma of live mice (**Fig 4B and 4C,** and S1 Movie). As a comparison, the motility coefficient of CXCR4 or integrin deficient B lineage cells is lower than 2 [5]. Finally, SOCS3 signaling was proposed to control FAK-mediated integrin adhesion in vitro [20]. In BM, B-lineage cell movement is strictly dependent on α4β1 integrin-mediated adhesion to VCAM-1, and reduced integrin-mediated adhesion alters B cell morphology from an amoeboid to a rounded cell shape [5]. Thus, to test if SOCS3 played any role in integrin mediated adhesion of B-lineage cells to the extracellular matrix in BM parenchyma we examined the amoeboid cell shape of SOCS3-deficient and sufficient B-lineage cells, as described [5]. Briefly, we measured the x and y length ratios of SOCS3-deficient and sufficient B cells in vivo and found that their cell shapes were undistinguishable (**Fig 4D**). These data demonstrate that SOCS3 signaling is not required for controlling B-lineage cell shape changes that occur during development as cells reduce integrin-mediated adhesion.

## Discussion

CXCL12 has been proposed to induce JAK/STAT tyrosine phosphorylation and to promote their association with CXCR4, and small molecule JAK inhibitors reduced CXCR4 function in vitro [27, 29–31]. In turn, shortly after engaging CXCL12, CXCR4 signaling through JAK2 and JAK3, and several STATs, also promoted SOCS3 expression in IM-9 cell line [27, 32]. Since these findings were reported several studies examined the interplay between SOCS3, JAKs and STATs in CXCR4 signaling and downstream biological activities. Increased SOCS3 expression by genetic manipulation or by treatment with cytokines, was found to repress CXCR4-mediated chemotaxis to CXCL12 in vitro, and to mobilize hematopoietic progenitors from BM [27, 33]. Conversely, conditional SOCS3 deficiency using the MMTV-cre approach, which promotes cre recombinase expression and recombination in some epithelial cells and in several hematopoietic cells including B-lineage cells, led to a significant accumulation of immature B cells in BM. It was proposed that SOCS3 negatively regulates FAK phosphorylation and integrin-mediated adhesion to the extracellular matrix [20]. However, other studies using primary cells and cell lines derived from JAK3-deficient patients, failed to detect any requirement for JAK2 and JAK3 signaling in CXCL12-mediated biological activities [34]. Furthermore, in mice conditionally deficient in SOCS3 specifically in B-lineage cells (using the same Igα promoter driving cre recombinase expression, *Mb1* ^*Cre/+*^) it was observed that developing B cell subsets were normally represented in BM and there was no evidence for an accumulation of late stage immature B cell subsets [21].

In this study, we demonstrate that immature B cell egress from BM into peripheral lymphoid organs is fully intact in the absence of B-cell intrinsic SOCS3 expression. Furthermore, we also found that CXCR4 internalization, migration towards CXCL12, and adhesion to the extracellular matrix, as measured by their amoeboid cell shape when adherent to the BM parenchyma [5], is independent of SOCS3 signaling. We conclude that CXCR4 signaling in B-lineage cells in vitro, or in vivo under homeostatic conditions is independent of SOCS3 expression. Thus, our studies are in direct agreement with studies reported by Tarlinton and colleagues [21], and extend those findings by demonstrating that B-lineage cell shape and migration in vivo is independent of SOCS3 signaling. Our findings are also in agreement, albeit indirectly, with other studies showing no involvement of *Jak2* and *Jak3* in CXCR4 signaling [34]. The mechanistic explanation for such discrepancies could be at two levels, namely in vitro and in vivo. At the in vitro level, most studies showing direct association of CXCR4 with SOCS3, JAKs and STATs, used cell lines (mostly HEK293 and IM-9) that were treated with small molecule JAK antagonists that may have poorly understood off-target effects [27, 29, 30]. Furthermore, in some experiments, these cell lines were transduced with cDNAs encoding SOCS genes and JAKs, which may have resulted in their insertion into genomic locations controlling some CXCR4 functions [27, 29]. Although another study validated the JAK2 and CXCR4 association by co-immunoprecipitation using a Jak2-deficient cell line, the authors did not examine CXCR4-mediated biological activities (e.g. cell migration towards CXCL12, Ca^2+^ flux) in Jak2 deficient and sufficient cells [29]. At the in vivo level, one plausible reason of why Tarlinton and colleagues as well as our findings differ from findings reported by Silberstein and colleagues could be that differences may have occurred during mice breeding. For example, breeding of CD45.1 C56BL/6 mice from NCI and Tac led to a spontaneous mutation in *Sox13* leading to a selective deficiency in V**γ**4^+^ **γδ**T17 cells [35]. Similarly, a mutation in the guanine nucleotide exchange factor dedicator of cytokinesis 8 (DOCK8) occurred during breeding of NLRP10 deficient mice, which inadvertently caused a severe migratory defect in dendritic cells in vivo [36].

## Supporting Information

S1 Movie Intravital two-photon microscopy of developing B cell subsets in BM calvaria of *Socs3* ^*fl/fl*^ *Mb1* ^*Cre/+*^ *Rag1* ^*GFP/+*^ mice (left) and *Socs3* ^*+/+*^ *Mb1* ^*Cre/+*^ *Rag1* ^*GFP/+*^ (right) mice. Time-lapse imaging (39-μm-thick z stack) of BM calvaria shows movement of developing B cells (GFP+, green). BM blood vessels (red) were visualized by tetramethylrhodamine-conjugated dextran (molecular mass 2,000 kDa) injection i.v. Time is shown as hh:mm:ss. Scale bar is 50 μm.
(MP4)
